# IgD-λ POEMS syndrome presenting with edema, pulmonary hypertension, and renal impairment: A case report

**DOI:** 10.1097/MD.0000000000051433

**Published:** 2026-11-06

**Authors:** Jie Feng, Junle Zhao, Chao Song, Hongrui Cui, Qiongzhen Lin

**Affiliations:** a Department of Nephrology, The First Hospital of Hebei Medical University, Shijiazhuang, China.

**Keywords:** case report, glomerular microangiopathy-like lesions, IgD-λ subtype, POEMS syndrome, pulmonary hypertension

## Abstract

**Rationale:**

Polyneuropathy, organomegaly, endocrinopathy, monoclonal plasma-cell disorder, and skin changes (POEMS) syndrome is a rare multisystem syndrome associated with clonal plasma-cell disorders. The IgD-λ subtype is uncommon, and a low-level monoclonal protein may escape detection by routine electrophoresis and immunofixation electrophoresis (IFE), delaying diagnosis.

**Patient concerns:**

A 38-year-old woman was admitted in April 2025 with intermittent bilateral lower-extremity edema for >3 years, elevated serum creatinine for 8 months, and acute worsening over 4 days. She subsequently developed pulmonary hypertension, polyserous effusions, splenomegaly, lymphadenopathy, anemia, thrombocytosis, and renal impairment, and had been diagnosed elsewhere with idiopathic pulmonary arterial hypertension.

**Diagnoses:**

Right-heart catheterization confirmed precapillary pulmonary hypertension. Routine serum and urine protein studies, including IFE, were negative. Following multidisciplinary evaluation, nerve-conduction studies showed peripheral neuropathy; serum vascular endothelial growth factor (VEGF) was elevated; and bone marrow studies demonstrated a clonal plasma-cell population with cytoplasmic λ light-chain restriction. Extended serum IFE, including anti-IgD and anti-IgE antisera, detected a trace, nonquantifiable monoclonal IgD component, establishing IgD-λ POEMS syndrome. Renal biopsy showed POEMS-associated glomerular microangiopathy-like lesions with acute tubular injury.

**Interventions:**

The patient received dexamethasone acetate plus lenalidomide and underwent autologous stem-cell transplantation on December 2, 2025.

**Outcomes:**

By March 2026, peripheral edema had resolved, pulmonary hypertension was mild on echocardiography, serum creatinine had decreased to 60 μmol/L, and VEGF had normalized.

**Lessons:**

POEMS syndrome should be considered in patients with unexplained multisystem disease, particularly edema or polyserous effusions accompanied by pulmonary hypertension and renal impairment. Negative routine M-protein testing should not exclude the diagnosis; VEGF testing, bone marrow evaluation, and extended IFE may reveal an occult plasma-cell clone.

## 1. Introduction

POEMS syndrome (polyneuropathy, organomegaly, endocrinopathy, monoclonal plasma-cell disorder, and skin changes) is a rare multisystem paraneoplastic syndrome driven by a clonal plasma-cell disorder. It may manifest with polyneuropathy, organomegaly, endocrinopathy, skin changes, extravascular volume overload, and hematologic abnormalities.^[1,2]^ Because of its marked clinical heterogeneity and broad multisystem involvement, diagnosis is frequently delayed. A monoclonal plasma-cell disorder is a mandatory diagnostic criterion; however, a low clonal burden or an occult monoclonal protein may result in negative routine protein electrophoresis and immunofixation electrophoresis (IFE), increasing diagnostic difficulty.^[1,3]^ The intact immunoglobulin heavy chain in POEMS syndrome is usually IgA or IgG, whereas the IgD-λ subtype is rare.^[4]^ We report a case of IgD-λ POEMS syndrome with an occult M-protein, initially presenting with extravascular volume overload and subsequently developing precapillary pulmonary hypertension and renal impairment, and discuss its clinicopathologic features and a potential mechanism of endothelial injury.

## 2. Materials and methods

### 2.1. Ethics and patient consent

This retrospective single-case report was approved by the Clinical Research Ethics Review Committee of The First Hospital of Hebei Medical University (Approval No. [2026] Research Ethics Review No. S01101, approved on August 10, 2026). Written informed consent was obtained from the patient for publication of clinical data, laboratory results, pathological images, and relevant clinical figures.

## 3. Case presentation

### 3.1. Patient information

A 38-year-old woman was admitted in April 2025 because of intermittent bilateral lower-extremity edema for more than 3 years, elevated serum creatinine (SCr) for 8 months, and worsening symptoms for 4 days. More than 3 years before admission, bilateral lower-extremity edema developed without an apparent precipitant, but she did not seek medical attention. Eight months before admission, the edema worsened. At another hospital, SCr was 93.7 μmol/L, and echocardiography demonstrated pericardial effusion and pulmonary hypertension, with an estimated pulmonary artery systolic pressure (PASP) of approximately 54 mm Hg. Her edema improved after diuretic and antihypertensive therapy.

She subsequently presented to a tertiary hospital in Beijing because of progressively reduced exercise tolerance. Pulmonary function testing showed severely impaired diffusing capacity. Computed tomography pulmonary angiography demonstrated dilation of the main pulmonary artery, pericardial effusion, bilateral pleural effusions. Echocardiography estimated PASP at 77 mm Hg. Right-heart catheterization demonstrated precapillary pulmonary hypertension, with an mean pulmonary arterial pressure (mPAP) of 36 mm Hg, pulmonary arterial wedge pressure (PAWP) of 12 mm Hg, and pulmonary vascular resistance (PVR) of 2.7 Wood units. Abdominal ultrasonography showed splenomegaly. Laboratory testing showed hemoglobin of 102 g/L, platelet count of 385 × 10^9^/L, SCr of 98.6 μmol/L, blood urea nitrogen of 9.32 mmol/L, and N-terminal pro-B-type natriuretic peptide of 6455.1 pg/mL. Serum free λ and κ light chains were 264.0 and 80.5 mg/L, respectively, with a κ/λ ratio of 0.30. Serum IFE showed no monoclonal protein. Autoantibodies and tumor markers were unremarkable. Because the underlying cause of her precapillary pulmonary hypertension had not yet been recognized, she was initially considered to have idiopathic pulmonary arterial hypertension (PAH) and was treated with macitentan. Thereafter, she developed nausea, abdominal distension, and hypotension (100/70 mm Hg), and her SCr increased to 237.68 μmol/L; she was therefore transferred to our hospital.

### 3.2. Physical examination findings

On admission, vital signs were stable. She appeared anemic. Multiple small, soft, mobile, nontender lymph nodes were palpable in both axillae, the neck, and the groin. Hepatic percussion tenderness was present. The spleen was palpable 3 fingerbreadths below the left costal margin and was firm. Mild pitting edema was present in both lower limbs. Muscle strength was grade 5 in all extremities, and no pathologic reflexes were elicited.

### 3.3. Diagnostic assessment

Laboratory tests showed hemoglobin of 97 g/L, platelet count of 379 × 10^9^/L, SCr of 260.0 μmol/L, blood urea nitrogen of 30.02 mmol/L, B-type natriuretic peptide of 39 pg/mL, and complement C3 of 0.83 g/L. Routine urinalysis was unremarkable, with no hematuria or proteinuria. Routine serum and urine protein electrophoresis and IFE detected no monoclonal protein. C-reactive protein was 16.02 mg/L, procalcitonin was 2.83 ng/mL, tumor necrosis factor-α was 11.15 pg/mL (reference range, ≤5.6 pg/mL), interleukin-1β was 3.38 pg/mL (≤12.3 pg/mL), and interleukin-6 was 11.79 pg/mL (≤12 pg/mL). Baseline endocrine findings are provided in Table S1, Supplemental Digital Content 1. Blood was sampled on menstrual cycle day 7; follicle-stimulating hormone, luteinizing hormone, prolactin, testosterone, progesterone, and estradiol were within their respective follicular-phase reference intervals. Free triiodothyronine was 2.63 pmol/L and total triiodothyronine was 0.48 nmol/L, both below the reference range; free thyroxine, thyroid-stimulating hormone, and parathyroid hormone were normal. Serum cortisol was not measured.

Echocardiography showed moderate pulmonary hypertension, with an estimated PASP of 65 mm Hg, and a moderate pericardial effusion. Renal ultrasonography showed a left kidney measuring 11.6 × 4.6 cm and a right kidney measuring 9.9 × 4.6 cm, with abdominopelvic fluid. Chest computed tomography demonstrated partial atelectasis of the left lower lobe, a large pericardial effusion, chest-wall soft-tissue edema, and multiple small bilateral axillary lymph nodes. Acute kidney injury, precapillary pulmonary hypertension, polyserous effusions, anemia, splenomegaly, and lymphadenopathy were initially considered. Macitentan-related gastrointestinal adverse effects were considered to have reduced oral intake and effective circulating volume. After intravenous fluid replacement and supportive care, SCr decreased to 113.8 μmol/L, supporting a prerenal contribution to the acute kidney injury.

Because of cardiovascular, respiratory, hematologic, and renal involvement in the setting of negative routine monoclonal-protein studies, a multidisciplinary team evaluated the patient for a plasma-cell disorder. Bone marrow aspiration showed plasma cells accounting for approximately 6.0% of nucleated cells. Flow cytometry detected a clonal plasma-cell population accounting for 2.16% of nucleated cells, with the phenotype CD38^+^, CD138^+^, CD19^−^, CD56^−^, CD34^−^, and CD20^−^ and cytoplasmic λ light chain restriction. Bone marrow biopsy showed increased plasmacytoid cells; immunohistochemistry was positive for CD138 and λ light chain and negative for κ light chain (Fig. 1).

**Figure 1.**
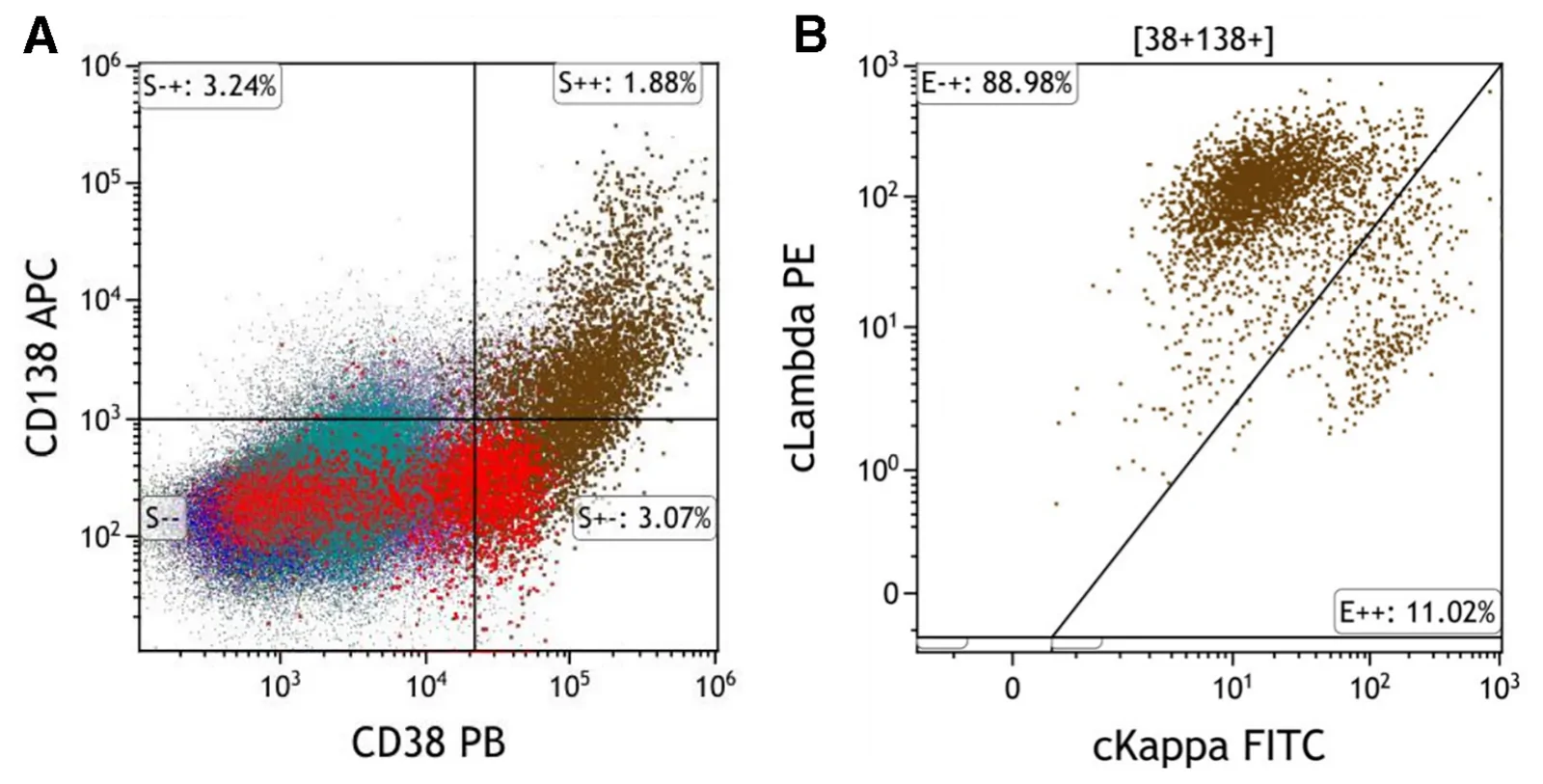
Bone marrow flow-cytometric analysis demonstrating a λ light chain-restricted clonal plasma-cell population. (A) CD38/CD138 analysis identifying the plasma-cell population. (B) Cytoplasmic κ/λ light chain analysis within the CD38^+^CD138^+^ plasma-cell gate, demonstrating cytoplasmic λ light-chain restriction. According to the final flow-cytometric report, clonal plasma cells accounted for approximately 2.16% of nucleated cells. APC = allophycocyanin, FITC = fluorescein isothiocyanate, PB = Pacific Blue, PE = phycoerythrin.

Serum and urine specimens were sent to the Institute of Hematology and Blood Diseases Hospital, Chinese Academy of Medical Sciences, for an M-protein identification panel. Extended serum IFE, including antisera against IgG, IgA, IgM, κ, λ, IgD, and IgE, showed no monoclonal component in the routine IgG, IgA, IgM, κ, or λ lanes; however, the IgD lane showed a monoclonal IgD component in the γ region. The serum M-protein concentration was reported as 0.00 g/L, indicating a trace, nonquantifiable component. Urine IFE detected no monoclonal protein (Fig. 2). Repeat serum free κ and λ light chains were 159.61 and 246.33 mg/L, respectively; urine free κ and λ light chains were 316 and 306 mg/L, respectively. Both light chains were elevated, whereas the κ/λ ratio was not clearly abnormal and therefore could not independently establish clonality.^[5]^ Serum vascular endothelial growth factor (VEGF) was 769.85 pg/mL (reference range, 9.25–142 pg/mL). Nerve-conduction studies showed reduced motor conduction velocities and amplitudes with prolonged distal latencies in the bilateral tibial and common peroneal nerves, as well as reduced sensory conduction velocities in the bilateral superficial peroneal nerves. Funduscopic examination revealed bilateral papilledema. Whole-body positron emission tomography/computed tomography (PET/CT) showed multiple retroperitoneal, para-aortic, and bilateral inguinal lymph nodes without abnormal fluorodeoxyglucose uptake. In view of polyneuropathy, a λ light chain-restricted clonal plasma-cell disorder, elevated VEGF, splenomegaly, extravascular volume overload, and papilledema, IgD-λ POEMS syndrome was diagnosed.

**Figure 2.**
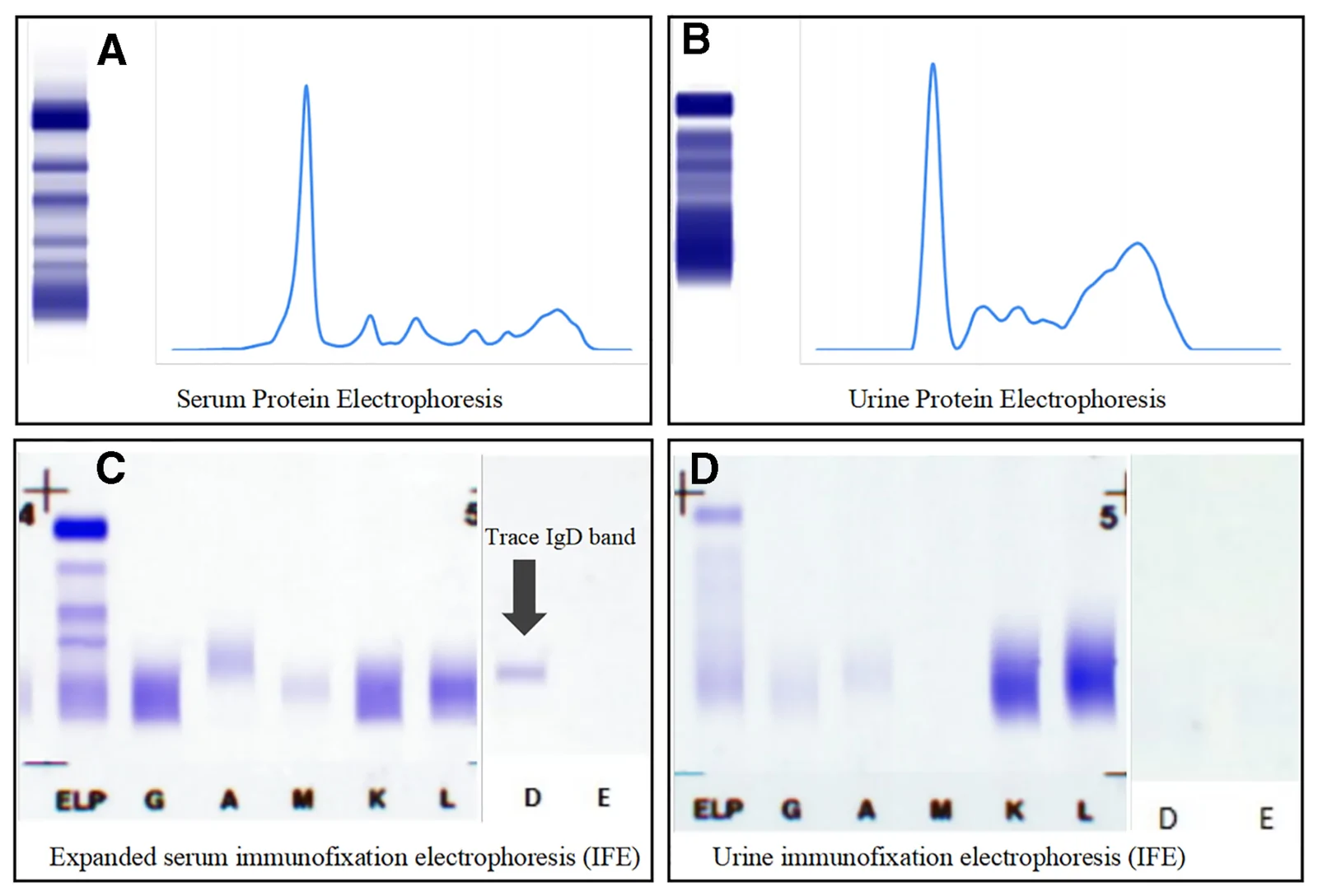
Serum and urine protein studies. (A) Serum protein electrophoresis showed no quantifiable monoclonal spike. (B) Urine protein electrophoresis showed no discrete monoclonal peak. (C) Extended serum IFE, including anti-IgD and anti-IgE antisera, detected a trace, nonquantifiable monoclonal IgD component (arrow). (D) Urine IFE showed no monoclonal component. ELP, protein electrophoresis; G, IgG; A, IgA; M, IgM; K, κ light chain; L, λ light chain; D, IgD; E, IgE. IFE = immunofixation electrophoresis.

Kidney biopsy was performed to define the renal lesion (Fig. 3). Light microscopy showed 38 glomeruli, 1 of which was ischemically sclerotic. The remaining glomeruli exhibited hypertrophic capillary loops, intracapillary neutrophil infiltration and karyorrhectic debris, diffuse endothelial swelling, basement-membrane thickening, and segmental double-contour formation. Tubular epithelial cells showed severe vacuolar and granular degeneration with focal brush-border loss. Renal arterioles displayed wall thickening with hyalinosis, fibrous intimal hyperplasia and sclerosis, and luminal narrowing. Immunofluorescence microscopy showed no specific staining for immunoglobulins, complement components, or κ and λ light chains, with no evidence of immune-complex or monoclonal immunoglobulin deposition. Electron microscopy showed marked widening of the lamina rara interna containing flocculent material and cellular debris, endothelial swelling with capillary luminal narrowing, segmental podocyte foot-process effacement, and prominent tubular epithelial vacuolar degeneration with increased lysosomes and partial microvillar loss. The pathologic diagnosis was POEMS-associated glomerular microangiopathy-like lesions with acute tubular injury; the latter was considered possibly related to infusion of hypertonic human albumin.

**Figure 3.**
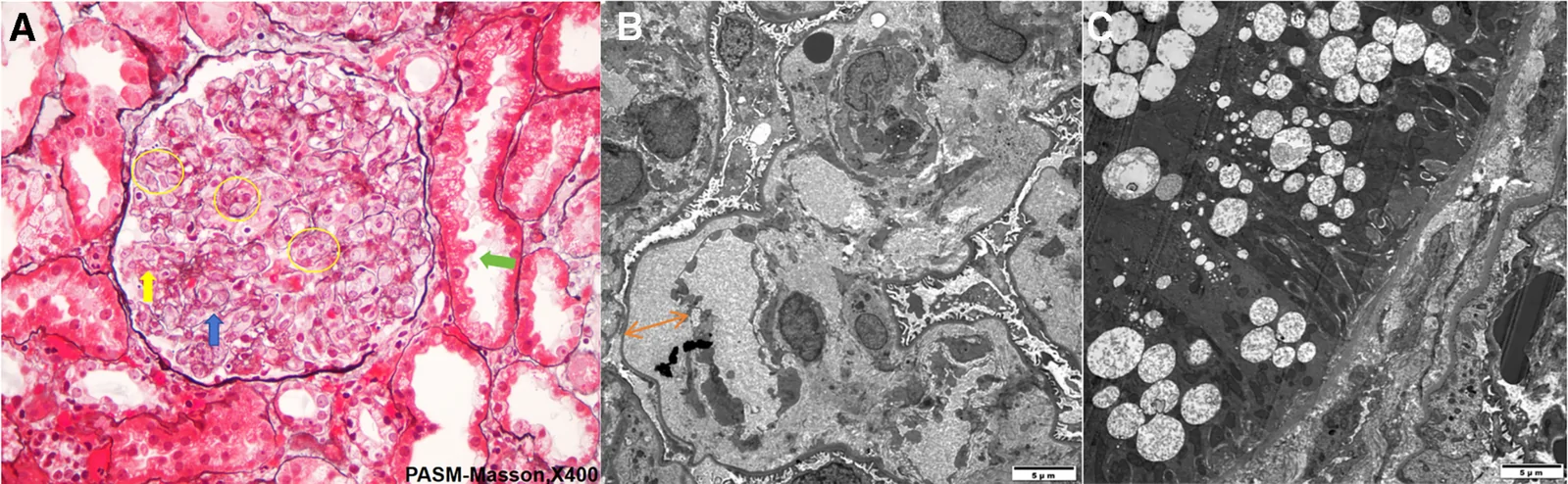
Renal biopsy findings. (A) PASM–Masson staining (×400) showed glomerular microangiopathy-like changes. Yellow circles indicate glomerular endothelial swelling; the yellow arrow indicates segmental double-contour formation of the glomerular basement membrane; the blue arrow indicates capillary luminal narrowing; and the green arrow indicates tubular epithelial vacuolar degeneration. (B) Electron microscopy showed marked widening of the lamina rara interna (subendothelial space; orange arrows), accompanied by endothelial swelling and capillary luminal narrowing. (C) Electron microscopy showed prominent tubular epithelial vacuolar degeneration, increased lysosomes, and partial microvillar loss, consistent with acute tubular injury. PASM = periodic acid-silver methenamine.

### 3.4. Treatment and follow-up

After diagnosis, oral dexamethasone acetate (39.75 mg, equivalent to 53 tablets of 0.75 mg each, once weekly) plus oral lenalidomide (10 mg once daily for 21 days followed by 7 days off) was administered for 3 months. Oral aspirin 100 mg once daily was given concurrently. By July 2025, her clinical symptoms had improved and SCr had decreased to 68 μmol/L. She underwent autologous stem-cell transplantation on December 2, 2025. At follow-up in March 2026, peripheral edema had resolved, echocardiography suggested improvement to mild pulmonary hypertension, SCr was 60 μmol/L, and serum VEGF was 84 pg/mL (reference value, <160 pg/mL). The clinical course is summarized in Figure 4.

**Figure 4.**
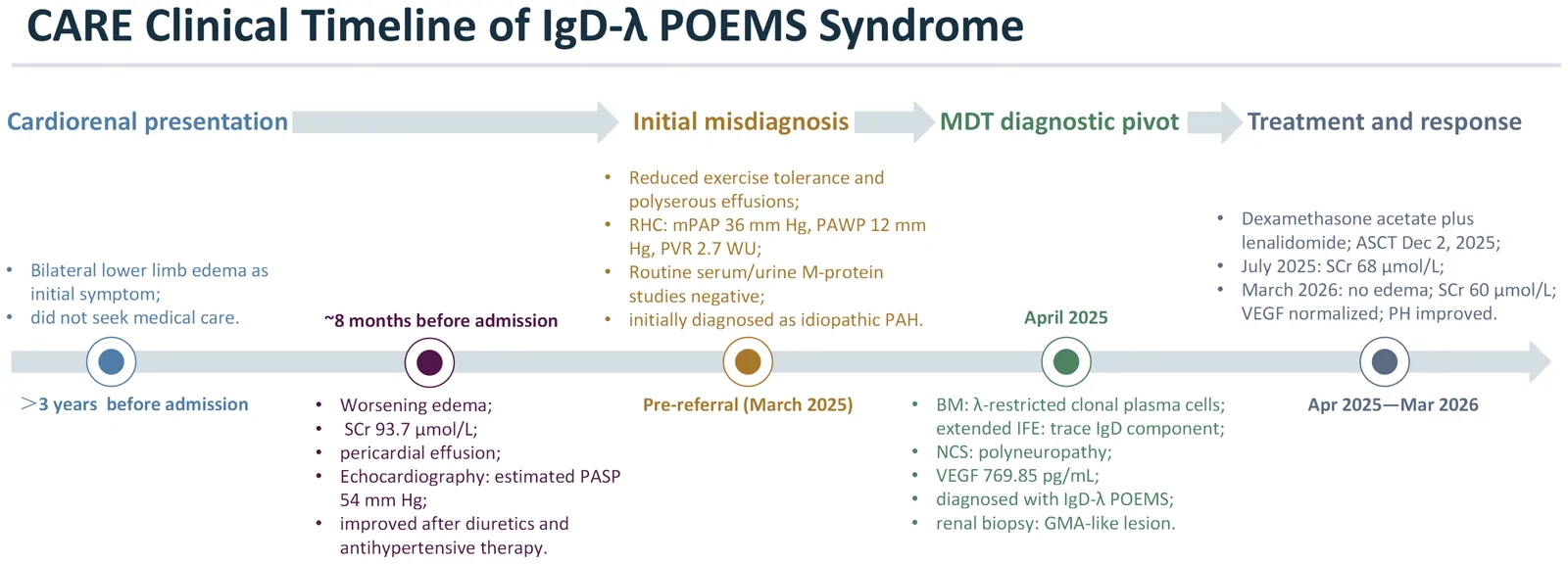
Timeline of the patient’s clinical course. The timeline summarizes events from symptom onset through diagnosis, treatment, and follow-up. In April 2025, extended serum immunofixation electrophoresis (IFE) detected a trace monoclonal IgD component, and bone marrow examination identified a λ light chain-restricted plasma-cell clone, supporting the diagnosis of IgD-λ POEMS syndrome. ASCT = autologous stem-cell transplantation, IFE = immunofixation electrophoresis, MDT = multidisciplinary team, NCS = nerve-conduction studies, PASP = pulmonary artery systolic pressure, POEMS = polyneuropathy, organomegaly, endocrinopathy, monoclonal plasma-cell disorder, and skin changes, PVR = pulmonary vascular resistance, RHC = right-heart catheterization, SCr = serum creatinine, VEGF = vascular endothelial growth factor.

## 4. Discussion

The diagnosis of POEMS syndrome requires both mandatory criteria, polyneuropathy and a monoclonal plasma-cell disorder, together with at least 1 other major criterion and 1 minor criterion.^[1,2]^ In this case, diagnosis was delayed because the plasma-cell clone was occult. POEMS syndrome usually has a low clonal burden and a small M-protein concentration; consequently, routine protein electrophoresis and immunofixation electrophoresis (IFE) may be negative.^[1,3]^ The IgD-λ isotype further increased this diagnostic challenge. In a cohort of 154 patients with POEMS syndrome and pulmonary hypertension, IgA-λ, IgG-λ, and λ light chain-only patterns accounted for 67%, 21%, and 12%, respectively, whereas no IgD isotype was reported.^[4]^ Physiologically, IgD represents approximately 0.25% of serum immunoglobulins, has a half-life of approximately 2.8 days, and contains a protease-susceptible long hinge region.^[6]^ Thus, a low-level IgD clone may not produce a recognizable M-protein peak; omission of anti-IgD antiserum from routine IFE panels further increases the risk of false-negative results. In our patient, only extended serum IFE detected a trace, nonquantifiable IgD component. Consistent with this observation, mass-spectrometry assays detected M-proteins in 20 of 21 patients previously classified as having nonsecretory POEMS syndrome, including IgD-λ cases.^[7]^

Therefore, negative routine M-protein testing should not exclude POEMS syndrome when the clinical phenotype is compelling. Extended serum IFE incorporating anti-IgD, anti-IgE, κ, and λ antisera should be combined with serum and urine free light-chain testing, bone marrow aspiration/biopsy, flow cytometry, immunohistochemistry, electrophysiologic testing, VEGF measurement, and imaging for osteosclerotic lesions or plasmacytoma.^[2,3]^ Immunoprecipitation-based mass spectrometry may be useful when routine and extended IFE remain negative.^[7]^

The initial diagnosis of idiopathic PAH reflected the nonspecific presentation and occult clone. Right-heart catheterization established precapillary pulmonary hypertension, but this hemodynamic phenotype alone did not establish idiopathic PAH. Edema, polyserous effusions, renal impairment, and reduced exercise tolerance could initially be attributed to cardiorenal dysfunction or right-heart failure, while peripheral neuropathy was clinically inconspicuous. Negative routine serum and urine M-protein studies further reduced suspicion for POEMS syndrome. Recognition of multisystem involvement, including splenomegaly, papilledema, elevated VEGF, and a λ-restricted plasma-cell clone, ultimately led to reattribution of the pulmonary hypertension to POEMS syndrome.

Renal involvement is not a diagnostic criterion for POEMS syndrome, but glomerular microangiopathy-like lesions are characteristic pathologic findings.^[8,9]^ The endothelial swelling, capillary luminal narrowing, and double-contour formation in our patient were consistent with this pattern. VEGF provides a plausible pathobiologic link between the renal and pulmonary manifestations. In the kidney, VEGF-related endothelial injury may contribute to glomerular microvascular damage; in the pulmonary circulation, VEGF and inflammatory cytokines may promote endothelial dysfunction, small-arterial remodeling, and precapillary pulmonary hypertension.^[3,8–11]^ Although causality cannot be established from a single case, the parallel improvement in edema, pulmonary hypertension, renal function, and VEGF after clone-directed therapy and autologous stem-cell transplantation (ASCT) supports the reversibility of POEMS-related endothelial injury.^[12–14]^ This case highlights the value of early multidisciplinary assessment and extended M-protein testing in patients with unexplained multisystem disease.

## 5. Conclusions

IgD-λ POEMS syndrome is extremely rare and may yield negative results on routine M-protein testing because of a low circulating monoclonal IgD concentration, its short half-life, and incomplete coverage of conventional test panels. POEMS syndrome should be considered in patients with unexplained edema, polyserous effusions, precapillary pulmonary hypertension, renal impairment, and other multisystem abnormalities. Negative routine testing does not exclude the diagnosis. VEGF measurement, electrophysiologic testing, bone marrow evaluation, extended serum IFE incorporating anti-IgD and anti-IgE antisera, and urine IFE are important complementary investigations. Kidney biopsy may define the renal lesion and provide pathologic evidence for POEMS-associated endothelial injury.

## Author contributions

**Methodology:** Chao Song, Hongrui Cui.

**Writing – original draft:** Jie Feng, Junle Zhao.

**Writing – review & editing:** Qiongzhen Lin.


