# Supplementary material for: IgD-λ POEMS syndrome presenting with edema, pulmonary hypertension, and renal impairment: A case report
Source: Medicine (Baltimore). 2026 Nov 6;105(45):e51433. doi: 10.1097/MD.0000000000051433 (PMC13747426; doi:10.1097/MD.0000000000051433)
Supplement: Supplementary file 1 [file medi-105-e51433-s001.docx]

**Supplementary Table S1. Baseline endocrine laboratory findings**

Blood samples were collected on 29 April 2025. The patient’s last menstrual period was 23 April 2025, with a usual cycle length of 30 days; the gonadal hormone panel was obtained on cycle day 7.

| **Analyte** | **Result** | **Unit** | **Institution-specific reference interval** | **Interpretation** |
| --- | --- | --- | --- | --- |
| Free triiodothyronine (FT3) | 2.63 | pmol/L | 3.53–7.37 | Below reference interval |
| Free thyroxine (FT4) | 8.68 | pmol/L | 7.98–16.02 | Within reference interval |
| Thyroid-stimulating hormone (TSH) | 5.81 | mIU/L | 0.56–5.91 | Within reference interval |
| Total triiodothyronine (TT3) | 0.48 | nmol/L | 0.92–2.38 | Below reference interval |
| Fasting plasma glucose | 5.77 | mmol/L | 3.9–6.1 | Within reference interval |
| Parathyroid hormone | 50.2 | pg/mL | 12–88 | Within reference interval |
| Follicle-stimulating hormone (FSH) | 7.02 | mIU/mL | Follicular phase: 3.85–8.78 | Within follicular-phase interval |
| Luteinizing hormone (LH) | 6.68 | mIU/mL | Follicular phase: 2.12–10.89 | Within follicular-phase interval |
| Prolactin | 24.51 | ng/mL | 3.34–26.72 | Within reference interval |
| Testosterone | 0.10 | ng/mL | 0.10–0.90 | Within reference interval |
| Progesterone | 0.32 | ng/mL | Follicular phase: 0.31–1.52 | Within follicular-phase interval |
| Estradiol | 15.43 | pg/mL | Follicular phase: 15.16–148.13 | Within follicular-phase interval |
| Serum cortisol | Not assessed | — | — | Not assessed |

Note: Reference intervals are those established by the Clinical Laboratory, The First Hospital of Hebei Medical University.

a.Sex hormone results were interpreted using follicular-phase reference intervals because sampling was performed on cycle day 7.

b.Serum cortisol was not measured during the diagnostic evaluation.
